# **Retraction notice for:** “Suppressive effect of platycodin D on bladder cancer through microRNA-129-5p-mediated PABPC1/PI3K/AKT axis inactivation” [Braz J Med Biol Res 2021;54(3): e10222]

**DOI:** 10.1590/1414-431X2022e10222retraction

**Published:** 2022-08-15

Dayin Chen^1,2^ https://orcid.org/0000-0002-6810-0521, Tingyu Chen^3^ https://orcid.org/0000-0001-6339-5503, Yingxue Guo^1^ https://orcid.org/0000-0003-1238-6767, Chennan Wang^1^ https://orcid.org/0000-0001-6846-1805, Longxin Dong^1^ https://orcid.org/0000-0002-6383-6476, and Chunfeng Lu^1,3^ https://orcid.org/0000-0002-1978-3266

^1^Department of Pharmacology, Basic Medical College, Jiamusi University, Jiamusi, Heilongjiang, China

^2^Department of Urology, the First Affiliated Hospital of Jiamusi University, Jiamusi, Heilongjiang, China

^3^School of Medicine, Huzhou University, Huzhou, Zhejiang, China

Retraction for: Braz J Med Biol Res | doi: 10.1590/1414-431X202010222 | PMID: 33470388 | PMCID: PMC 7814303

The authors requested retraction of the article “Suppressive effect of platycodin D on bladder cancer through microRNA-129-5p-mediated PABPC1/PI3K/AKT axis inactivation” that was published in volume 54 no. 3 (2021) (Epub January 15, 2021) in the Brazilian Journal of Medical and Biological Research.

The corresponding author stated the following: “In a subsequent experiment, we repeated the experiment in this manuscript. Unfortunately, we had different results than before. This led to inaccurate conclusions in this manuscript”.

In addition, the manuscript was questioned by independent journalists from the “PubPeer” website <https://pubpeer.com/publications/51FACD700BCEB2DE71D4748E96BE72>. This denouncement consisted of potential data falsification and/or inaccuracy.

Thus, the Editors decided to retract this article to avoid further damage to the scientific community. The Brazilian Journal of Medical and Biological Research remains vigilant to prevent misconduct and reinforces the Journal’s commitment to good scientific practices.

